# Supplementary material for: Spatial and temporal clustering of typhoid fever in an urban slum of Dhaka City: Implications for targeted typhoid vaccination
Source: PLoS Negl Trop Dis. 2024 Jun 24;18(6):e0012273. doi: 10.1371/journal.pntd.0012273 (PMC11226105; doi:10.1371/journal.pntd.0012273)
Supplement: Attachment — Submitted filename: 2023 Dec PLoS NTD Spatial Typhni Dhaka .docx [file pntd.0012273.s001.docx]

PLOS Neglected Tropical Diseases

Spatial and Temporal Clustering of Typhoid Fever in an Urban Slum of Dhaka City: Implications for Targeted Typhoid Vaccination

Dec 5, 2023

General comments

67-68: This sentence is a bit confusing as worded; what ‘high-resolution data’ is missing—are you saying the data needed to inform an analysis like this, or that analyses using high-resolution data are missing? The way it is worded suggests the former, but I think you mean the latter?

75: Name the study being analysed (or cite it); its not clear until the end of the first paragraph of the methods if this paper is doing a new primary typhoid fever surveillance study to answer this question around spatial /temporal clustering, or if you aare using results from another major study to answer this question. It should just be made a little clearer earlier on. In addition to naming the study in your last paragraph of intro, I might move the “Full details of the study have been published elsewhere [12]” to the second sentence of the study population paragraph, and then follow that with “In brief, approximately 158,000….”

83: what kind of health centers (inpatient/outpatient) helps frame

89: All typhoid cases that occurred in the study area… as identified by the ‘burden of enteric fever at three urban sites…’ study passive surveillance sites? I am confused by how the cases in the follow up period were identified? The 172 originally available to be chosen as index cases, is all the cases that were identified in the study area via the 7 health centers in the original study? How were the 268 follow up cases identified? Presumably all these cases were retrospectively identified in original study?

How were ring sizes determined? 50m interval (or even 200m are small areas) but I could imagine it being relevant in densities such as Mirpur.

134: What is the population density overall? How large is the study area, overall in size? Can you describe living situation a little more—formal vs informal housing with no piped water, apartment complexes, etc.

144-153: This paragraph is hard to follow; you are citing lots of numbers and lots of use of the word ‘respectively’ and its hard to follow. Where are the incidence numbers for intermediate spatial cluster for instance? You have these in a table, so you don’t have to list each and every one here, you can highlight the overall trends (and 1 or 2 of the most important)—or find another way to succinctly show this in a way that is easier to follow.

156: Are these ‘progressively larger’ or just comparisons to outer concentric circles? Can you say this in a way that summarizes for readers; incidence was highest in the inner most circles, relative to the more distant concentric circles (a few numbers, and then reference the figure)- -same comment applies to many of these paragraphs. The data aren’t presented in a completely linear way (mostly you show innter most circle, then outermost, then concentric. But why this order? Can you describe the pattern so the reader doesn’t have to decipher it, and then back it up with the numbers instead?)

171: I think this is not right, “Over a period of 20 months, we observed 141 typhoid cases”--- didn’t you have more cases then that but you excluded? Again im not sure I get how these are different then the ones identified in follow up.

175: I think this should be re-worded; I read this as saying that IRR was not different between case and control clusters at the outer concentric circles. That’s not true—IRR’s are still like 3. Youre trying to say that the IRR’s are a bit lower than the inner most circle. Please reword this

There needs to be more in the discussion. The first paragraph of the discussion reads a little more like how the results should actually be (or, at best, is just a re-hash of those results). In discussion, it would be good to talk more about:

1) What do you think explains these data results—spatial and temporal
2) What hypotheses of transmission are supported or not supported by these findings
3) How these results may be extrapolated to other settings, or not extrapolated, based on what you know of this area versus other typhoid endemic areas.
4) Are these cases mostly sporadic, clusters, outbreaks? Are any of these household cases? Do you know?
5) More about reactive vaccination campaigns (perhaps needs to be more in background too)—it’s a leap to just show results and then say this in a conclusion without any discussion or further support. How do each of your conclusions (spatial, and temporal) each independently impact recommendations? Does lack of temporal difference mean that reactive vaccination doesn’t have to be immediate? That disease risk is more about specific, highly focal areas with specific environmental risks, or about risk generated following individual cases, or what?

Figures:

Figure 2: might be better as a bar graph given these are biweekly results. Are these the start dates or end dates of each period. Each figure should include captions that stand on its own (Number of typhoid cases as reported by study xxxxx, within the study catchment area in Mirupr, Dhaka…)

Figures should have acronyms spelled out in the caption (if not in figure/table), e.g., IQR, IRR, SD, etc. of each figure
