# Supplementary material for: Spatial and temporal clustering of typhoid fever in an urban slum of Dhaka City: Implications for targeted typhoid vaccination
Source: PLoS Negl Trop Dis. 2024 Jun 24;18(6):e0012273. doi: 10.1371/journal.pntd.0012273 (PMC11226105; doi:10.1371/journal.pntd.0012273)
Supplement: Attachment — Submitted filename: Typhoid space-time clustering_responses.docx [file pntd.0012273.s002.docx]

10 March 2024

Dr. Shaden Kamhawi, Dr. Paul Brindley, & Peter J. Hotez

Editors-in-Chief

PLOS Neglected Tropical Diseases

Re: Response to comments of the reviewers on the manuscript, “Spatial and Temporal Clustering of Typhoid Fever in an Urban Slum of Dhaka City: Implications for Targeted Typhoid Vaccination” (PNTD-D-23-01352)

Dear Dr. Shaden Kamhawi, Dr. Paul Brindley, & Peter J. Hotez,

On behalf of all co-authors, I am submitting a revised manuscript reflecting the comments and suggestions made by reviewers on the manuscript entitled “Spatial and Temporal Clustering of Typhoid Fever in an Urban Slum of Dhaka City: Implications for Targeted Typhoid Vaccination”.

We thank the reviewers for providing detailed and insightful feedback on the analysis and report. Below, we have provided point-by-point responses to the questions and comments brought forth by the reviewers and have referenced the manuscript where changes have been made.

We believe that these revisions have strengthened the manuscript and that the analytic approaches and main findings are now more clearly communicated.

We are grateful for the opportunity to be published in the PLOS Neglected Tropical Diseases journal. I am available to respond to any additional questions or points of clarification regarding this submission.

Sincerely,

Faisal Ahmmed

**Point by point response to the referee comments**

**Key Review Criteria: Required for Acceptance?**

**Reviewer #1:** The authors have clearly reported the methodology including the study setting, selection of cases and matched controls and marking the geographical areas around index cases and controls. Sample size estimation is not reported.

**Response:** This analysis is derived from a dataset obtained from a large, cluster-randomized trial. Consequently, a sample size calculation tailored to a specific hypothesis test was not conducted. However, the statistical power of our analysis given the study sample has been included in the methods section (Lines 191-197).

**Reviewer #2:** The authors aimed to provide further understanding of transmission of typhoid fever linked to index case identified in a high-risk setting, a slum in this study and over a time frame that the authors defined. The objectives are clear and the population under study has been described sufficiently to answer the question under investigation. The authors investigated clusters of typhoid transmission linked to an index case and also to index controls.

**Reviewer #3:**

- Ethical approvals and consent processes are in reference 12, but typically they should be explicitly stated in the manuscript.

**Response:** The institutional ethical approval and consent processes have been added on page (lines 132-134)

- How were controls approached, recruited, and enrolled? Please briefly elaborate on the process.

**Response:** Baseline information on all censused populations in the study catchment area was collected during the census, and clinical data of patients enrolled in passive surveillance were also gathered (Meiring JE, et al, Lancet Global Health 2021 [REF 12]). We utilized both datasets retrospectively for this analysis and outlined the process of selecting cases and their corresponding controls in the "Selection of Index Cases and Index Controls" section (lines 137-154)

- Per PNTD checklist, please explicitly state the study objectives and include a clearly stated hypothesis.

**Responses:** The study research question has now been clearly stated in lines 81-84.

- Since the STRATAA study (ref 12) serves as the backbone of the study population, a brief summary of that study would be helpful in Background for appropriate context.

**Response:** A brief summary of the STRATAA study has been added to the method section on page (lines 108-118).

- Study population: The study population described in Lines 77-87 appears different from that in ref 12. This study cites ~158,000 residents versus ~110,000 residents in ref 12 Figure 1. This study surveys from August 2016 through April 2018 (I believe it is from Sept 2016 based on Fig 2) versus passive surveillance dates in ref 12 from Jan 1, 2017 – Dec 31 2018. This study identified 172 total cases during the 20-month study period (Line 130) versus 359 S. Typhi cases in Dhaka over 24 different months in ref 12 Figure 1. Please either describe the study population, or explain any differences compared with ref 12, especially with regard to census population and study dates as these numbers greatly impact the case counts and statistical analyses on incidence and incidence ratios.

**Response:** The noted differences in denominators and typhoid cases between the reference paper (#12) and this analysis are due to the selection of slightly different study start dates (this analysis counted cases from 15 August 2016 versus 01 January 2017 in the reference paper) as well as the factoring of migration, births, and deaths (dynamic population) in this analysis versus a static population in the published study.

- Lines 84-86: Please briefly elaborate on the sample acquisition protocols and “microbiological culture methods.” I see reference #12, but some basic details should be present in the Methods section. For example, were two sets of bloods routinely drawn? Was blood culture performed by an automated blood culture machine – if so, what machine? How was S. Typhi isolated and identified (broths, agars, slants, biochemical strips, agglutination, PCR, sequencing, etc.)?

**Response:** A brief description of the identification of typhoid patients has been added on page (lines 123-130)

- Line 89: Were the same typhoid cases that were used as index cases then counted in relation to another index case? Were any cases counted more than once?

**Response:** Yes, typhoid cases that occurred within the spatiotemporal cluster of an index case were subsequently assessed as an index case. This is stated on Line 185-187.

- Line 105: Please offer a brief biological (or other) rationale for selecting time windows of 0-14 vs 15-28 days post infection. What do these windows represent or what epidemiological questions can they be expected to answer?

**Response:** The temporal categories used in this analysis roughly capture the period for one transmission event (estimated based on average incubation period for typhoid fever is 14 days). Greater temporal resolution was limited by our sample size and extending the period of observation beyond 28 days increases the likelihood that cases occurring within the time period were introduced through separate transmission pathways. We have included this limitation in the discussion section (Page, Line 332-338).

- The manuscript would greatly benefit from a map (or maps by Ward) of the geographic region with points to indicate the index and control households and surrounding ring of analysis. Added layers for waterways, water piping, relevant sewerage, and/or topographical boundaries could be especially useful as well. These details are essential for a reader to visualize the spatial and temporal clustering analysis. Regarding human subject confidentiality, typically if large markers are used, if the map is zoomed far enough out, and if there is a high background density of people (5,000-10,000 people within any given 200m radius), the identity of individuals can remain adequately preserved.

**Response:** Figure 1 has been replaced with a geographic map of the study area depicting households of the study population. The figure provides an illustration of the population evaluated in spatial rings surrounding a sample case and two controls as all typhoid cases in a period of 28 days following enrollment of the index case (21/10/2027)

- Why did the authors select the method of choice? Did you consider a simple point-pattern cluster or density analysis of all households over the study period to further investigate clustering? Or a space-time scan to evaluate temporal-spatial relationships? Arcgis and satscan softwares have a lot of powerful options.

**Response:** Our analysis uses a simplistic approach that aims at evaluates whether we observe an increased risk of typhoid in the geographic region immediately surrounding an index case and, in the period, immediately following identification. We acknowledge the availability of powerful spatio-temporal tools that utilize advanced process to generate complex associations, however given the data available and the simplicity of the question being asked, we selected a more traditional epidemiologic approach.

**Reviewer #4:** Methods are generally clear and straightforward. Some more direct formal comparisons of statistical difference/power would be nice.

**Response:** A statistical power calculation has been incorporated into the statistical methods section on page (lines 191-197).

**Key Review Criteria: Results**

**Reviewer #1:** All-important results are clearly narrated. Tables and figures are adequate.

**Reviewer #2:** The authors have clearly presented the results. The figures and the Tables are clear and of good quality. The results clearly indicate increased transmission in areas in close proximity to the index case and therefore case area targeted interventions would be useful. I have a few questions and also comments for the authors to consider in the discussion

- In line 100 under 'Selection of cases and controls' the authors indicate that cases could also be resampled as controls. How many of the cases were resampled as controls if any, and how does this resampling affect the assertion that the population density was higher in cases than it was in controls?

**Response:** In our analysis, no cases were subsequently selected as controls. The reviewer highlights an important point regarding the substantive difference in population density in case clusters compared to control clusters. This baseline difference, given the potential relationship between population density and disease transmission, has now been discussed in the limitations section (line 338-340).

- Figure 2 shows the number of typhoid cases observed bi-weekly during study period. There is an increase in the number of cases identified from August 2017. Is this increase reflected in both controls and case clusters? What factor may have resulted in the increased observations and also how does this factor affect spatial clusters?

**Response:** Controls were sampled contemporaneously (i.e., matched at time of identification), therefore control enrolment frequency mirrors the case numbers. We required that spatial clusters did not overlap each other. During periods of higher enrolment, it is possible that random selection of controls was artificially pushed towards areas with fewer cases, leading to minor bias in our analysis.

- Under limitations the authors have indicated the 28-day duration as not sufficient to assess transmission dynamics. Perhaps the 200M radius may also not be optimal to observe a change in transmission. According to TABLE 2 typhoid cases were identified along the radius regardless of spatial size in both cases and controls.

**Response:** We agree with the limitations of the geographic boundaries used to delineate the spatial clusters assessed in this analysis. This limitation is discussed as the first limitation in lines 316-321.

**Reviewer #3:**

- Line 131: Were any of the 31 cases occurring within 28 days before study end included as case counts if they fell 200m from an index case occurring 28 days before study end? I ask because 31 cases in one month is a sizable burden of disease when 172 total cases over 20 months averages to ~8.6 cases per month. Did an outbreak occur? Were these 31 cases close to one another or on a common piped water grid?

**Response:** Our data show potentially 4 outbreaks, the largest of which occurred in the last month of the study. In the final month, of 31 cases, X occurred within the 200m radius geographic cluster surrounding an index case. Although further epidemiologic investigation on potential transmission pathways and risk factors was not conducted nor the sequencing of isolated pathogens to investigate genetic relatedness, our data suggest that an outbreak did occur and that it was unlikely to be due to seasonality.

- Line 132: How was the incidence of 0.44 cases per 100,000 person-days calculated? By my calculation, if 141 cases occurred over 20 months in a population of 158,000 people, the incidence per 100,000 person-days should be: 141 cases / (158000 persons * (20 months * 365 days / 12 months)) * 100000 = 0.15 cases per 100,000 person-days. Please share how you calculated incidence.

**Response:** The incidence rate was calculated by dividing the number of cases by the person-time within the follow-up period. In Table 2, for the case clusters with r=0-200m, we observed 355 (87+268) cases within the 28-day follow-up among 80,098,678 (42,520,780+37,577,898) person-days. The total person-time was calculated accounting migration, death, or outcome. The incidence rate was then calculated by dividing 355 cases by 80,098,678 person-days, multiplied by 100,000, resulting in approximately 0.44 cases per 100,000 person-days.

- Lines 134-135: Cluster population size and density were substantially higher in case clusters (Table 1). Given the mechanisms of typhoid transmission, would this difference confound your results? Please address this in Discussion.

**Response:** The impact of substantive baseline differences in population density between case and control clusters has been included in the discussion. (lines 338-340) Although control clusters were selected randomly, since we restricted spatial overlap of control clusters and since typhoid occurs in densely populated areas, it is possible that control clusters were systematically selected in less densely populated areas. We did not control for population density in the analysis and therefore difference in population density may lead to residual confounding of our results.

- Line 139: 268 observed cases is above the 141 cases reported during the study period. Were cases counted multiple times? If so this should be explained in methods. A map of points and geometric rings as requested above would also assist in visualizing this. Does this impact your analysis?

**Response:** As described in Lines 185-187. We observed that case and person time redundancy occurred in both index case and control cluster simultaneously. And we believe that incidence rate and incidence rate ratio were not affected by this redundancy. However, we have addressed this issue as a limitation in the discussion on page (lines 329-332). A map of geometric rings has been provided in Fig-1.

- Figure 2: X-axis suggests the study is “from Sept 2016 through Apr 2018.” Versus “from August 2016” as is stated in in Line 79.

**Response:** The figure has now been corrected (Fig 2).

- I do not see a table that matches Line 112, “To evaluate temporal clustering, we measured the IR of typhoid fever in cluster residents (r=200m) during the first 14 days after selection […]” Table 4 is the closest, but makes comparisons at 50m increments, rather than r=200m. Was there a comparison of all cases from 0-200m at 0-14d vs 15-28d?

**Response:** The sentence has been revised, and the result now aligns with the information presented in Table 4.

**Reviewer #4:** Results are not clearly presented.

**Response:** The results section has been rewritten to improve clarity (lines 202-275).

**Key Review Criteria: Conclusions**

**Reviewer #1:** The study conclusion is in line with the study objective and main findings.

**Reviewer #2:** Conclusions are supported by the data presented and the public health relevance is clear as regards case area targeted interventions including vaccines as authors recommend but also other interventions should be considered.

**Reviewer #3:**

- The discussion section needs improvement. A scholarly discussion with references on the meaning and significance of the results is necessary. Contextualize the study within the local typhoid dynamics (and literature). Relate the findings to other studies on the spatial and temporal nature of typhoid.

**Response:** The discussion has been revised to reflect the reviewer’s recommendations.

- Lines 180-188: Other limitations include use of household coordinates (which may not equate to the place where typhoid was transmitted/acquired), counting of cases multiple times (if they fall into multiple 200m radii), and perhaps a lack of other epidemiologic data (including household level data of cases, familial relationships, water/sewage data, knowledge of carriers in the area, etc.)

**Response:** These limitations have been added to the discussion in lines 329-332 and 338-340.

- Line 188: Change “transmission events,” to incident patterns or similar. The study is not presently designed to evaluate transmission patterns.

**Response:** This sentence has been revised (line 334)

- Lines 190-194: These three sentences seem to repeat the data summarized in lines 171-176. If they do not add information, they can probably be removed to avoid redundancy.

**Response:** The paragraph has been removed (lines 347-356).

- Lines 195-196: Similar to the comment above for Line 188 wh, I don’t think the data supports a transmission observation so much as the existence of co-incident cases occurring near one another.

**Response:** We have revised this statement as well (line 352).

- Lines 196-200: “Reactive vaccination” and “case-area targeted intervention (CATI) strategies” are introduced for the first times in the penultimate sentence of the manuscript. If a discussion point, please define and elaborate your idea. Why do you think the data supports a “reactive vaccination” campaign as the best intervention strategy? Why not mass vaccination campaigns? Or routine/EPI immunizations? More discussion is warranted with appropriate references.

**Response:** The Case Area Targeted Intervention (CATI) approach could be advantageous for controlling diseases where vaccine stockpiles are limited. Unlike mass vaccination campaigns, CATI focuses on specific geographic areas or communities with high disease incidence or transmission rates. By targeting these high-risk areas, CATI can effectively control disease outbreaks with low vaccine coverage, optimizing the utilization of available vaccine doses. We support administration of typhoid vaccines through mass vaccination and routine EPI immunization programmes however, in settings where national typhoid programs have yet to be implemented or where vaccine supply is limited, we recommend this interim approach to maximize vaccine impact in the most vulnerable populations. We have revised the conclusion based on this comment (lines 306-314)

**Reviewer #4:** Conclusions are not well supported by data presented-- more is needed in discussion.

**Response:** We have revised the conclusion section.

**Editorial and Data Presentation Modifications?**

**Reviewer #1:** The authors are advised to report the sample size estimation and also to further enhance the discussion section.

**Response:** We have added the sample size estimation on page (lines 191-197)

**Reviewer #2:** (No Response)

**Reviewer #3:**

- Line 57: comma after “S. Typhi”
- Line 62: disease should be plural
- Line 67: the word “and” may be erroneous or some words are missing
- Line 83: Are the 7 participating healthcare centers inclusive of ALL the healthcare centers with blood culture capability serving the ~158,000 residents? If not, then the population surveilled may be less.
- Line 87: “typhi” should be capitalized
- Lines 99-101: Were any index controls selected more than once?
- Line 142: missing a space
- Line 152: missing a space
- Line 157: missing an open parenthesis
- Lines 175-176: seems contradictory
- Lines 190-194: Redundant to lines 171-178. Not adding any additional discussion.
- References formatting is irregular. See the way that authors are listed, for example

**Response:** Thank you for pointing out the errors in the manuscript and we have corrected all these carefully.

**Reviewer #4:** My main concern is that there needs to be more in the discussion. The first paragraph of the discussion reads a little more like how the results should actually be (or, at best, is just a re-hash of those results). In discussion, it would be good to talk more about:

- What do you think explains these data results—spatial and temporal.

**Response:** We have endeavoured to expand our discussion section based on our observations from the analysis. We observed the highest incidence of typhoid in the innermost circle (50m radius), with incidence gradually decreasing with increasing spatial distance. However, we did not observe any temporal patterns of disease decreasing within 28 days from disease onset. Maybe 28 days of follow-up not sufficient to measure the temporal clustering of typhoid incidence.

- What hypotheses of transmission are supported or not supported by these findings.

**Response:** Typhoid transmission may correlate with other environmental factors, but the distance of population living could be proxy factor of typhoid transmission.

- How these results may be extrapolated to other settings, or not extrapolated, based on what you know of this area versus other typhoid endemic areas.

**Response:** Although this analysis has some limitations, it provides insight into the spatiotemporal relationship of typhoid fever in endemic settings, which could be further explored in more comprehensive studies. The evidence generated from this study might be generalizable, but careful consideration is needed for local adaptation based on the unique characteristics of each typhoid endemic area.

- Are these cases mostly sporadic, clusters, outbreaks? Are any of these household cases? Do you know?

**Response:** The study was conducted in an urban slum of Dhaka, Bangladesh, and no significant outbreak reported during the study period. Result support to spatial clustering. Five out of 8 household had developed typhoid after infected. Please see in below table.

| Sl. | HHID | Date of Onset | Household transmission |
| --- | --- | --- | --- |
| 1 | D031270079 | 11/4/2017 | Within 3 days |
|  | D031270079 | 11/7/2017 |  |
| 2 | D031270248 | 12/4/2017 | No |
|  | D031270248 | 2/16/2018 |  |
| 3 | D031400177 | 3/29/2018 | Within 1 day |
|  | D031400177 | 3/30/2018 |  |
| 4 | D031490010 | 3/23/2018 | Within 10 days |
|  | D031490010 | 4/3/2018 |  |
| 5 | D051930112 | 2/3/2018 | Within 1 day |
|  | D051930112 | 2/4/2018 |  |
| 6 | D052360064 | 10/29/2017 | No |
|  | D052360064 | 3/5/2018 |  |
| 7 | D052440077 | 4/4/2017 | No |
|  | D052440077 | 6/9/2017 |  |
| 8 | D052540413 | 10/30/2017 | Within 16 days |
|  | D052540413 | 11/15/2017 |  |

- More about reactive vaccination campaigns (perhaps needs to be more in background too)—it’s a leap to just show results and then say this in a conclusion without any discussion or further support. How do each of your conclusions (spatial, and temporal) each independently impact recommendations? Does lack of temporal difference mean that reactive vaccination doesn’t have to be immediate? That disease risk is more about specific, highly focal areas with specific environmental risks, or about risk generated following individual cases, or what?

**Response:** The entire paper is designed to examine the disease burden in a case-targeted area based on spatiotemporal cluster without any intervention. We observed a significant spatial clustering of typhoid but failed to provide evidence temporal clustering within a 28-day period. We believe that if vaccination is conducted in an infected area as early as possible after any infection, the spread of the disease will be controlled, and temporal clustering would become apparent. This idea of reactive vaccination is now discussed in the discussion section, specifically in lines 356-361.

.

**Key Review Criteria: Summary and General Comments**

**Reviewer #1:** This is an important study with sufficient rigors and novelty. Data analysis is adequately performed. Sample size estimation is missing which should be included. Further, discussion is inadequate and recommend strengthening this section by adequate literature review of similar studies, and interpretation of study findings especially policy implications.

**Response:** Sample size section is added in lines 191-197.The discussion has been revised (lines 277-365).

**Reviewer #2:** The risk of transmission of typhoid fever from a case to those around is a well known event. This research work brings in actionable intelligence in particular case area targeted intervention strategies. It would have been more valuable to describe and compare the risk factors in the case and control spatial clusters, but also increase the observation time. However this study provides a platform for even more robust studies to provide high resolution in the transmission dynamics, and inform precisely the optimal areas to receive intervention and over what period.

**Response:** In future analysis, we will explore the difference in risk factors between case and control spatial clusters using the baseline characteristics of all censused populations. We will also plan to analyse with increased observation time (lines 332-338).

**Reviewer #3:** Summary:

Ahmmed et al. analyzed passive surveillance data from August 2016 to April 2018 of ~158,000 residents in two wards of Mirpur, an urban slum area of Dhaka City, Bangladesh, collected in the Strategic Typhoid Alliance across Africa and Asia (STRATAA) studies, to evaluate for spatial and/or temporal clustering of typhoid fever case households. Index cases were identified, control households >200m away were selected from the census area, and typhoid cases were enumerated over 28 days. Incidence was compared across increasing radii in 50m increments as well as between the first two and second two weeks following an index case. The authors determined that incidence (risk) is highest in the 50m surrounding an index case household and risk is approximately stable between the first two weeks and the second two weeks. The authors recommend reactive vaccination based on the results.

**General comments:**

- The major strengths of this study are its basis on the highly detailed STRATAA study, the use of a strong microbiology infrastructure afforded by icddr,b, and the accuracy of a census performed every 6 months to inform incidence data. The major weaknesses are a lack of details/clarity in the methods, a lack of a map/graphical image to help understand the study population and incidence in context, and a lack of clear scholarly discussion of the work. I believe the article could be strengthened and more meaningful by addressing the above critiques.

**Response:** We have added one map showing a graphical representation of case and control clusters and study population in Fig 1. Since a map including typhoid incidences over the study period is difficult due to overlapping issue of typhoid cases, so we did not attempt to plot any map for the incidence in context.

**Reviewer #4:** This is a nice, simple paper with interesting findings that add an important component to literature about typhoid that adds value to public health and epidemiologic understanding of Typhi transmission. However, key details are missing to help maximize value, and the discussion is too sparse to support the conclusions. The results are also not clearly written, making it hard to follow the main themes of the results. With more details added throughout to delineate what this is/isnt (metods/results to help us understand what was done), and substantially more added to discussion, this paper could be a valuable contribution.

**Response:** Feedback from reviewers have been reflected in a revised manuscript. We hope that these modifications improve the clarity and depth of the paper.

**More specific comments**

- 67-68: This sentence is a bit confusing as worded; what ‘high-resolution data’ is missing—are you saying the data needed to inform an analysis like this, or that analyses using high-resolution data are missing? The way it is worded suggests the former, but I think you mean the latter?

**Response:** This phrase has been updated for clarity (lines 93-96).

- 75: Name the study being analysed (or cite it); its not clear until the end of the first paragraph of the methods if this paper is doing a new primary typhoid fever surveillance study to answer this question around spatial /temporal clustering, or if you aare using results from another major study to answer this question. It should just be made a little clearer earlier on. In addition to naming the study in your last paragraph of intro, I might move the “Full details of the study have been published elsewhere [12]” to the second sentence of the study population paragraph, and then follow that with “In brief, approximately 158,000….”

**Response:** We have provided clarification that this analysis evaluates an existing dataset that is part of the STRATAA consortium. References and a brief description have been included in lines 109-111.

- 83: what kind of health centers (inpatient/outpatient) helps frame

**Response:** Characteristics of the health centers included in the surveillance sites have been inserted in lines 119-121.

- 89: All typhoid cases that occurred in the study area… as identified by the ‘burden of enteric fever at three urban sites…’ study passive surveillance sites? I am confused by how the cases in the follow up period were identified? The 172 originally available to be chosen as index cases, is all the cases that were identified in the study area via the 7 health centers in the original study? How were the 268 follow up cases identified? Presumably all these cases were retrospectively identified in original study? How were ring sizes determined? 50m interval (or even 200m are small areas) but I could imagine it being relevant in densities such as wMirpur.

**Response:** Yes, we utilized the Bangladesh dataset from the multi-site study as mentioned (lines 108-109). In our analysis, we retrospectively utilized both census and passive surveillance data, considering a different study period for surveillance compared to the original published paper. A total of 172 cases of typhoid fever were identified during the study period. We have defined the spatial and temporal cluster in line 155-163 and the evaluated the cases within these spatiotemporal clusters in lines 169-178. The issue of case redundancy is addressed in the discussion section (lines 329-332).

- 144-153: This paragraph is hard to follow; you are citing lots of numbers and lots of use of the word ‘respectively’ and its hard to follow. Where are the incidence numbers for intermediate spatial cluster for instance? You have these in a table, so you don’t have to list each and every one here, you can highlight the overall trends (and 1 or 2 of the most important)—or find another way to succinctly show this in a way that is easier to follow.

**Response:** We revised this section to make it clear to understand (lines 233-243).

- 156: Are these ‘progressively larger’ or just comparisons to outer concentric circles? Can you say this in a way that summarizes for readers; incidence was highest in the inner most circles, relative to the more distant concentric circles (a few numbers, and then reference the figure)- -same comment applies to many of these paragraphs. The data aren’t presented in a completely linear way (mostly you show innter most circle, then outermost, then concentric. But why this order? Can you describe the pattern so the reader doesn’t have to decipher it, and then back it up with the numbers instead?)

**Response:** We have already reported the highest incidence observed in the innermost circle (lines 234-235197-198), and here we are trying to conclude that the incidence of typhoid in case index clusters decreases with increasing spatial distance from the population living in those areas. The sentence has been revised (lines 250-253).

- 171: I think this is not right, “Over a period of 20 months, we observed 141 typhoid cases”--- didn’t you have more cases then that but you excluded? Again im not sure I get how these are different then the ones identified in follow up.

**Response:** This sentence has been corrected (lines 279-281).

- 175: I think this should be re-worded; I read this as saying that IRR was not different between case and control clusters at the outer concentric circles. That’s not true—IRR’s are still like 3. Youre trying to say that the IRR’s are a bit lower than the inner most circle. Please reword this

**Response:** This sentence has been rephrased for clarity, please see line 283-285

**Main critique:**

There needs to be more in the discussion. The first paragraph of the discussion reads a little more like how the results should actually be (or, at best, is just a re-hash of those results). In discussion, it would be good to talk more about:

- What do you think explains these data results—spatial and temporal

**Response:** We have endeavoured to expand our discussion section based on our observations from the analysis. We observed the highest incidence of typhoid in the innermost circle (50m radius), with incidence gradually decreasing with increasing spatial distance. However, we did not observe any temporal patterns of disease decreasing within 28 days from disease onset. Maybe 28 days of follow-up not sufficient to measure the decrease of disease incidence.

- What hypotheses of transmission are supported or not supported by these findings

**Response:** Typhoid transmission may correlate with other environmental factors, but the distance of population living could be proximal factor of typhoid transmission.

- How these results may be extrapolated to other settings, or not extrapolated, based on what you know of this area versus other typhoid endemic areas.1

**Response:** Although this analysis has some limitations, it provides insight into the spatiotemporal relationship of typhoid fever in endemic settings, which could be further explored in more comprehensive studies. The evidence generated from this study might be generalizable, but careful consideration is needed for local adaptation based on the unique characteristics of each typhoid endemic area

- Are these cases mostly sporadic, clusters, outbreaks? Are any of these household cases? Do you know?

**Response:** The study was conducted in an urban slum of Dhaka, Bangladesh, and no significant outbreak reported during the study period. Result support to spatial clustering. Five out of 8 household had developed typhoid after infected. Please see in below table.

| Sl. | HHID | Date of Onset | Household transmission |
| --- | --- | --- | --- |
| 1 | D031270079 | 11/4/2017 | Within 3 days |
|  | D031270079 | 11/7/2017 |  |
| 2 | D031270248 | 12/4/2017 | No |
|  | D031270248 | 2/16/2018 |  |
| 3 | D031400177 | 3/29/2018 | Within 1 days |
|  | D031400177 | 3/30/2018 |  |
| 4 | D031490010 | 3/23/2018 | Within 10 days |
|  | D031490010 | 4/3/2018 |  |
| 5 | D051930112 | 2/3/2018 | Within 1 days |
|  | D051930112 | 2/4/2018 |  |
| 6 | D052360064 | 10/29/2017 | No |
|  | D052360064 | 3/5/2018 |  |
| 7 | D052440077 | 4/4/2017 | No |
|  | D052440077 | 6/9/2017 |  |
| 8 | D052540413 | 10/30/2017 | Within 16 days |
|  | D052540413 | 11/15/2017 |  |

- More about reactive vaccination campaigns (perhaps needs to be more in background too)—it’s a leap to just show results and then say this in a conclusion without any discussion or further support. How do each of your conclusions (spatial, and temporal) each independently impact recommendations? Does lack of temporal difference mean that reactive vaccination doesn’t have to be immediate? That disease risk is more about specific, highly focal areas with specific environmental risks, or about risk generated following individual cases, or what?

**Response:** The entire paper is designed to examine the disease burden in a case-targeted area based on spatiotemporal cluster without any intervention. We observed a significant spatial clustering of typhoid but failed to provide evidence temporal clustering within a 28-day period. We believe that if vaccination is conducted in an infected area as early as possible after any infection, the spread of the disease will be controlled, and temporal clustering would become apparent. This idea of reactive vaccination is now discussed in the discussion section, specifically in lines 356-361

**Figures:**

Figure 2: might be better as a bar graph given these are biweekly results. Are these the start dates or end dates of each period. Each figure should include captions that stand on its own (Number of typhoid cases as reported by study xxxxx, within the study catchment area in Mirupr, Dhaka…) Figures should have acronyms spelled out in the caption (if not in figure/table), e.g., IQR, IRR, SD, etc. of each figure

**Response:** We have modified the figure based on the comment.
