# Supplementary material for: Spatial and temporal clustering of typhoid fever in an urban slum of Dhaka City: Implications for targeted typhoid vaccination
Source: PLoS Negl Trop Dis. 2024 Jun 24;18(6):e0012273. doi: 10.1371/journal.pntd.0012273 (PMC11226105; doi:10.1371/journal.pntd.0012273)
Supplement: Attachment — Submitted filename: Response to reviewers.docx [file pntd.0012273.s003.docx]

14 May 2024

Dr. Shaden Kamhawi, Dr. Paul Brindley, & Peter J. Hotez

Editors-in-Chief

PLOS Neglected Tropical Diseases

Re: Response to comments of the reviewers on the manuscript, “Spatial and Temporal Clustering of Typhoid Fever in an Urban Slum of Dhaka City: Implications for Targeted Typhoid Vaccination” (PNTD-D-23-01352)

Dear Dr. Shaden Kamhawi, Dr. Paul Brindley, & Peter J. Hotez,

On behalf of all co-authors, I am submitting a 2^nd^ time revised manuscript reflecting the comments and suggestions made by reviewers on the manuscript entitled “Spatial and Temporal Clustering of Typhoid Fever in an Urban Slum of Dhaka City: Implications for Targeted Typhoid Vaccination”.

We thank the reviewers for providing detailed and insightful feedback on the analysis and report. Below, we have provided point-by-point responses to the questions and comments brought forth by the reviewers and have referenced the manuscript where changes have been made.

We are grateful for the opportunity to be published in the PLOS Neglected Tropical Diseases journal. I am available to respond to any additional questions or points of clarification regarding this submission.

Sincerely,

Faisal Ahmmed

**Reviewer's Responses to Questions**

Key Review Criteria Required for Acceptance? As you describe the new analyses required for acceptance, please consider the following:

**Methods**

- Are the objectives of the study clearly articulated with a clear testable hypothesis stated?
- Is the study design appropriate to address the stated objectives?
- Is the population clearly described and appropriate for the hypothesis being tested?
- Is the sample size sufficient to ensure adequate power to address the hypothesis being tested?
- Were correct statistical analysis used to support conclusions?
- Are there concerns about ethical or regulatory requirements being met?

**Reviewer #2:** The authors have now provided extra clarity to the methods section

**Reviewer #3:** The authors comprehensively and adequately addressed prior methodologic concerns/questions. Very nice incorporation of revised Fig 1 to visualize the methods employed.

**Reviewer #4:** The population (and geography) continues to be incompletely described; A map showing the area, including where cases were and controls and underlying pop density would help. How large even is the study region? Very relevant because we are talking about geospatial analyses here. It is not clear to me if the exclusion criteria for controls are incompletely described, given differences in pop density surrounding controls (which coudl be random, but seem so different as to suggest a potential systematic reason).

**Responses:** Thank you for your comment. A map illustrating the study area, including population density, has been incorporated into the revised manuscript as Figure 1. The map showed the boundaries of the study area indicating the density of households. To illustrate the cluster ring sizes, spatial rings of a selected index case and associated controls are also shown. Incident typhoid cases occurring within 28 days following case and control selection are indicated on the map with a legend providing the date of onset.

Index controls were randomly selected from the census records, ensuring that they had been residing in the study area for at least 28 days before the date of selection and had not been tested positive for typhoid within 14 days prior to the date of selection. Additionally, controls were selected such that circular regions of radius 200m surrounding the index case and both matched index controls could not overlap (lines 130-138). There were no additional exclusion criteria for the selection of controls.

**Results**

- Does the analysis presented match the analysis plan?
- Are the results clearly and completely presented?
- Are the figures (Tables, Images) of sufficient quality for clarity?

**Reviewer #2:** The results are clearly presented

**Reviewer #3:** Analysis and results are clearly and completely presented in the revised manuscript.

**Reviewer #4:** In general, the results adequately describe what is proposed. An additional map to clarify study pop and methods would be helpful. There is one map on page 46 of the pdf, but it is not interpretable and has no captions and otherwise appears to provide very limited information. A simple map showing underlying population density, the study area, and where cases and controls (perhaps with 200m circles, if visually doable) were would probably be very helpful.

**Responses:** Thank you for the suggestion. We have added captions to the figures in the manuscript (lines 150-159). However, we acknowledge that the map was uploaded independently and was not adjusted to its corresponding place in the text. We apologize for the inconvenience you faced in understanding the map.

**Caption of the figure excerpt from the manuscript is as follows:**

**Fig 1 Geographic representation of the study area and household density.** A typhoid index case and two controls are illustrated and the population residing within concentric ring clusters of increasing diameters surrounding the index case and controls are represented. Incident typhoid cases occurring in the 28-day period following case and control selection are shown. Base map source: Esri, HERE, Garmin, USGS, Intermap, INCREMENT P, NRCan, Esri Japan, METI, Esri China (Hong Kong), Esri Korea, Esri (Thailand), NGCC, (c) OpenStreetMap contributors, and the GIS User Community. Shapefiles are created and updated by the STRATAA Bangladesh GIS team. <https://www.arcgis.com/apps/mapviewer/index.html?layers=3b93337983e9436f8db950e38a8629af>

**Conclusions**

- Are the conclusions supported by the data presented?
- Are the limitations of analysis clearly described?
- Do the authors discuss how these data can be helpful to advance our understanding of the topic under study?
- Is public health relevance addressed?

**Reviewer #2:** The conclusions discussed are supported by the findings from the study.

**Reviewer #3:** The authors expanded the discussion greatly and with added nuance. For example, the inclusion of "our analysis was not designed to distinguish direct transmission events" is important. An alternative explanation for the spatial clustering is that a source outbreak (e.g., contaminated food shared, or water reaching a small region) occurred and infected individuals develop typhoid at variable rates, with some occurring <14 days and others up to 30 days. The authors also greatly improved discussion of study limitations and context. Two final considerations regarding the recommendation for reactive vaccination:

1) Based on the data presented, the authors recommend CATI; however I am not convinced this is a perfect solution. This control method assumes that a targeted vaccination following identification of an index vaccine would prevent cases occurring presumably ~14-28 days after the index case. However, development of humoral and cellular-mediated immune protection sufficient to prevent infection following challenge with S. Typhi is at least several weeks, and some individuals receiving CATI vaccine may be incubatory cases from the same source of infection, not yet presenting with symptoms. This nuance is sufficiently reflected in the limitations discussed and does not necessarily need to be further elaborated upon.

2) The use of CATI without antecedent case/household investigations could obscure the ability for public health teams to identify chronic S. Typhi carriers using serological methods. For example, one proposed tool to detect chronic carriers (which may be a source and longterm reservoir of transmission in these high incident communities) is anti-Vi IgG antibody, among other serological markers of chronic infection. It might be beneficial to perform household investigations of incident cases and collect blood/stool specimens from potential chronic carriers PRIOR to vaccinating them with typhoid conjugate vaccine, which would induce high titers of anti-Vi IgG antibody in vaccinees, losing the ability to detect chronic carriers possessing uniquely high titers compared to household members.

I would leave it to the authors to consider the above in their recommendations for CATI approaches, but changes are not requisite as these are areas of ongoing research.

**Responses:** We really appreciate the reviewer’s suggestions. We prefer to mention the limitation of CATI approach in the discussion based on your first suggestion (lines 281-290).

**Reviewer #4:** No-- I don't think the conclusion is well supported by or discussed in depth in the paper. "In countries lacking mass national campaigns and routine immunization programs, evidence of geographic clustering of typhoid cases suggests the potential impact of typhoid reactive vaccination in the population immediately surrounding identified cases." How so? Is Bangladesh one of these countries? Why only countries lacking mass national campaigns? What is done now? What about the time needed to vaccinate and to then develop immunity? How does that relate to the limitations in timelines of this study? I understand what the authors are trying to say, and there is more here now then before, but I think this statement is overly broad, and there continues to not be a lot of discussion around these points. If this is a central conclusion (as it appears to be) there should be more around this is discussion if this is the conclusion. Otherwise, the conclusion should state only what is clearly supported by the evidence-which is not around efficacy of different vaccine strategies.

**Responses:** Thanks for your suggestion, the conclusion of the abstract has been revised now based on the suggestions in lines 52-54.

**Editorial and Data Presentation Modifications?**

- Use this section for editorial suggestions as well as relatively minor modifications of existing data that would enhance clarity. If the only modifications needed are minor and/or editorial, you may wish to recommend "Minor Revision" or "Accept".

**Reviewer #2:** None

**Reviewer #3:** Change "sever" to "several" (Methods > Study Population > Line 109 in complete PDF)

**Responses:** Thank you for your feedback. The word has been corrected on line 110.

**Reviewer #4:** 67-68: run-on sentence

**Responses:** Thank you for bringing this to our attention. We have already revised the sentence as suggested in lines 90-93.

68: This sentence, "A better understanding of transmission linked to an identified typhoid fever case in a slum setting is needed" does not describe what this paper tries to do. This paper is looking at risk associated with spatiotemporal factors, not transmission factors of individual cases.

**Responses:** Thank you for clarifying. This sentence has indeed been revised as per your suggestion in lines 92-93 of the updated version.

83: what kind of health center-inpatient or outpatient? Pharmacy?

**Responses:** The health center type mentioned in lines 112-113

**Summary and General Comments**

- Use this section to provide overall comments, discuss strengths/weaknesses of the study, novelty, significance, general execution and scholarship. You may also include additional comments for the author, including concerns about dual publication, research ethics, or publication ethics. If requesting major revision, please articulate the new experiments that are needed.

**Reviewer #2:** This is an important study that provides a platform for future studies designed to investigate transmission dynamics

**Reviewer #3:** Ahmmed et al. effectively present a simple spatial and temporal risk analysis of typhoid fever occurring in two wards of Mirpur, Dhaka City, Bangladesh. The revisions to the manuscript are comprehensive and addressed all my previous comments adequately.

**Reviewer #4:** Overall, the revisions have been very helpful and this is a much stronger paper. The increased delineation of methods and results have helped make the study much clearer. However, important concerns remain, including around the populations included for control/cases, as well as the conclusions reached that are not really discussed.

**Major issues:**

- A major difference between cases and controls is the density; these are marked differences. Why is it? This does not seem likely to be random - is it because of the exclusion criteria (non overlapping with cases), or some other reason? This should also be addressed in limitations.

**Responses:** We have included further discussion of the potential bias introduced by the difference in population density discussed in Lines 312-315.

- How does inclusion of household clusters, in the setting of few overall cases, bias these results?

**Responses:** Our clusters defined based on geographical distance. We, however, observed a significant clustering of typhoid among people living within 200m radius. The highest incidence observed within 0-50m clusters including infected household members and also observed a significant risk of typhoid excluding the infected household members in the clusters 51-100m, 101-150m, and 151-200m. We have adjusted the confidence interval and p-value using robust standard error considering the potential bias due to the geographical clustering (was mentioned in lines 178-181).

- A description of the household clusters (or otherwise directly associated cases) should be included. With only 141 cases over 20 months, it could easily be that one or two households with cases of say 5 each, would explain most of your results by heavily weighting your innermost circle multiple times (household case 1 would have all 4 other people in house count toward innermost incidence rate; household case 2 would then have case 1 and the other 3 people count; household case 3 would have case 1 and 2 and the other 2 people count, etc etc).

**Responses:** Thanks for your suggestions. We have observed geographical clustering and a sentence has been added in the result section (lines 202-203).

- Was a sensitivity analysis done excluding immediate household members/cases from analysis would strengthen your conclusion that a vaccine campaign should focus on close geography (rather than just immediate household members, as is currently often the recommendation in many places).

**Responses:** We acknowledge the importance of sensitivity analysis to strengthen our conclusions. While we did not explicitly exclude immediate household members in a separate sensitivity analysis, we extended our analysis to populations living within 51-100m, 101-150m, and 151-200m radii, where household members were not considered in those rings. This broader analysis revealed a significant risk of typhoid compared to the control population (Table 4), consistent with our main findings.

- No-- I don't think the conclusion is well supported by or discussed in depth in the paper. "In countries lacking mass national campaigns and routine immunization programs, evidence of geographic clustering of typhoid cases suggests the potential impact of typhoid reactive vaccination in the population immediately surrounding identified cases." How so? Is Bangladesh one of these countries? Why only countries lacking mass national campaigns? What is done now? What about the time needed to vaccinate and to then develop immunity? How does that relate to the limitations in timelines of this study? I understand what the authors are trying to say, and there is more here now then before, but I think this statement is overly broad, and there continues to not be a lot of discussion around these points. If this is a central conclusion (as it appears to be) there should be more around this is discussion if this is the conclusion. Otherwise, the conclusion should state only what is clearly supported by the evidence-which is not around efficacy of different vaccine strategies.

**Responses:** Thanks for your suggestion, the conclusion of the abstract has been revised now based on suggestions in lines 52-54.
