# Supplementary material for: Spatial and temporal clustering of typhoid fever in an urban slum of Dhaka City: Implications for targeted typhoid vaccination
Source: PLoS Negl Trop Dis. 2024 Jun 24;18(6):e0012273. doi: 10.1371/journal.pntd.0012273 (PMC11226105; doi:10.1371/journal.pntd.0012273)
Supplement: Attachment — Submitted filename: Letter.docx [file pntd.0012273.s004.docx]

05 June 2024

Dr. Shaden Kamhawi, Dr. Paul Brindley, & Peter J. Hotez

Editors-in-Chief

PLOS Neglected Tropical Diseases

Re: Response to comments of the reviewers on the manuscript, “Spatial and Temporal Clustering of Typhoid Fever in an Urban Slum of Dhaka City: Implications for Targeted Typhoid Vaccination” (PNTD-D-23-01352R2)

Dear Dr. Shaden Kamhawi, Dr. Paul Brindley, & Peter J. Hotez,

On behalf of all co-authors, I am submitting the revised manuscript reflecting the special requirement of journal made by editors on the manuscript entitled “Spatial and Temporal Clustering of Typhoid Fever in an Urban Slum of Dhaka City: Implications for Targeted Typhoid Vaccination”. Below, we have provided point-by-point responses to the requirements.

We thank the reviewers for comprehensive reviews and feedback. We are grateful for the opportunity to be published in the PLOS Neglected Tropical Diseases journal.

Sincerely,

Faisal Ahmmed

**Figure Files:**

**Response:** All the figures are checked by the Preflight Analysis and Conversion Engine (PACE) digital diagnostic tool.

**Data Requirements:**

**Response:** We have uploaded the minimal data into PLOS submission system. In addition, we have added a text in the manuscript body for accessing these data in lines 344-347.

**Reproducibility:**

**Response:** The trial protocol was published previously and assigned its own identifier (DOI). We have cited that number in the method section (lines 127-128)

**References**

**Response:** All the references have been checked and formatted by Vancouver style. We have added a protocol reference and the reference list have been revised based on the current order.

In Addition, we have revised the **abstract** to make it within 300 words (lines 24-61).
